# The development of cardiac surgery in West Africa-the case of Ghana

**DOI:** 10.4314/pamj.v9i1.71190

**Published:** 2011-06-06

**Authors:** Frank Edwin, Mark Tettey, Ernest Aniteye, Martin Tamatey, Lawrence Sereboe, Kow Entsua-Mensah, David Kotei, Kofi Baffoe-Gyan

**Affiliations:** 1 National Cardiothoracic Center, Korle Bu Teaching Hospital, P.O. Box KB 846, Accra-Ghana; 2 Walter Sisulu Pediatric Cardiac Center for Africa, Sunninghill Hospital, Cnr Witkoppen & Nanyuki Rd, Johannesburg, South Africa

**Keywords:** Cardiac surgery, open-heart surgery, congenital heart disease, rheumatic heart disease, cardiopulmonary bypass

## Abstract

West Africa is one of the poorest regions of the world. The sixteen nations listed by the United Nations in this sub-region have some of the lowest gross domestic products in the world. Health care infrastructure is deficient in most of these countries. Cardiac surgery, with its heavy financial outlay is unavailable in many West African countries. These facts notwithstanding, some West African countries have a proud history of open heart surgery not very well known even in African health care circles. Many African health care givers are under the erroneous impression that the cardiovascular surgical landscape of West Africa is blank. However, documented reports of open-heart surgery in Ghana dates as far back as 1964 when surface cooling was used by Ghanaian surgeons to close atrial septal defects. Ghana's National Cardiothoracic Center is still very active and is accredited by the West African College of Surgeons for the training of cardiothoracic surgeons. Reports from Nigeria indicate open-heart surgery taking place from 1974. Cote D'Ivoire had reported on its first 300 open-heart cases by 1983. Senegal reported open-heart surgery from 1995 and still runs an active center. Cameroon started out in 2009 with work done by an Italian group that ultimately aims to train indigenous surgeons to run the program. This review traces the development and current state of cardiothoracic surgery in West Africa with Ghana's National Cardiothoracic Center as the reference. It aims to dispel the notion that there are no major active cardiothoracic centers in the West African sub-region.

## Background

Very few West African countries have the resources to provide optimum cardiac care to their populations. Cardiac (and to a lesser extent general thoracic) surgery requires relatively sophisticated diagnostic and surgical techniques and high level infrastructure operated by personnel with advanced training and expertise. The manpower and infrastructure required is largely unavailable on the African continent. The availability of cardiac surgeons per million of the population in North America and Europe is more than ten times the figure for Africa [1]. The treatment costs related to diagnosis and treatment of heart disease is way beyond the means of the largely indigent population in West Africa. In parts of western Africa, only 20% of the parents of children less than 15 years old requiring surgery for congenital heart disease are able to finance the operation within 12 months of diagnosis [2]. Large numbers of patients in West Africa lack the resources to access whatever treatment is available locally. The few citizens who can afford cardiac surgery resort to seeking treatment abroad. Apart from South Africa and Egypt with several active cardiac centers, the rest of the continent is still grappling with large populations with limited options for home-based cardiac surgery. Kenya and Sudan have active cardiac surgery centers but sub-optimal national economies in these countries still create major obstacles that limit the potential of these cardiac teams.

Against the backdrop of the lack of cardiovascular infrastructure in Africa, cardiovascular teams starting out in African countries are often misled into thinking that their programs are the first in their regions [3,4] or only the third after South Africa and Egypt [5]. With the pervasive lack of health infrastructure in Africa, such an erroneous view is perhaps not surprising but the fact remains that cardiac surgery, even open heart surgery is being practiced in several African nations apart from South Africa and Egypt.

This review was prompted by recent publications [3, 4] based on misconceptions of the history and current state of cardiac surgery in West Africa. It is our aim to point out significant developments on the cardiac surgery landscape of West Africa with Ghana's National Cardiothoracic Center as the point of reference.

## Origins of open heart surgery in West Africa

To our knowledge, four of the sixteen nations in the West African sub-region have documented accounts of open heart surgery. Recently, reports from Cameroon have indicated that this country is also making efforts to reach their citizens with open heart surgery locally [3, 4].

At the University of Nigeria Teaching Hospital (UNTH) in Enugu, the first open-heart surgery in Nigeria was performed on 1^st^ February 1974 [6]. The team of surgeons included M. Yacoub, F.A. Udekwu, D.C. Nwafor, C.H. Anyanwu, and others. By the year 2000, a total of 102 such operations had been carried out at the center by different Nigerian teams, with Professor Martin Aghaji's team being in the forefront [6]. Most of the cases were due to rheumatic heart disease, congenital heart disease, and diseases of the aorta. At the time of Eze and Ezemba's report [6], three government-owned centers were practicing open heart surgery in Nigeria, the UNTH group being the most active. The cardiac program in Nigeria unfortunately has not fared very well in terms of growth in patient numbers. A number of reasons have been put forward to account for the decline in open heart procedures in Nigeria – economic difficulty, political instability, corruption and mismanagement, poor funding, and others [6].

As far back as 1983, the group in La Cote D'Ivoire reported their results of the first 300 cases of open heart surgery performed in Abidjan for cardiac valve disease (149 cases), congenital heart disease (100 cases), endomyocardial fibrosis (40 cases) and other lesions [7]. The hospital mortality was 13.3%, mostly due to the severity of the condition prior to surgery. By 1987, this group published data on 851 open heart operations performed at the same institution in Abidjan [8]. Professor H. Yagni-Angate's team has continued the work of this group till recent times. Economic difficulty and civil strife in recent times contributed to the decline of the open heart program in Abidjan.

In Senegal, Professor M. Ndiaye's team at the Thoracic and Cardiovascular Surgery Department of Dakar′s Fann University Teaching Hospital began moves toward an open heart program in 1990 [9]. It took time, collaboration with several non-governmental organizations and especially a fortuitous encounter in 1995 with an American surgeon before the first open-heart operation took place in Senegal. In 1995, four patients underwent open heart surgery at the Aristide Le Dantec Hospital in Dakar, with a large US team taking part. Fifteen years later open-heart surgery has become common in this country of nearly 13 million, and Ndiaye′s department has become a reference point for francophone West Africa. They now have a permanent team performing open-heart operations regularly, 15-20% of cases being children [9].

**Figure 1:**
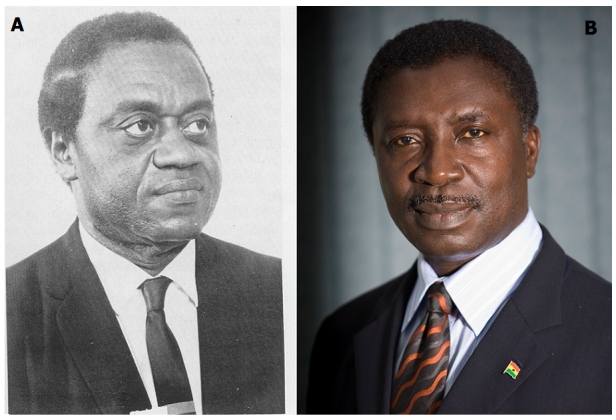
(A) Professor Charles Odamtten Easmon, pioneer of open-heart surgery in Ghana-(B) Professor Kwabena Frimpong-Boateng, founder of Ghana's National Cardiothoracic Center

In Ghana, our experience with open heart surgery began in 1964 when Professor C.O Easmon's team successfully performed closure of an atrial septal defect using surface cooling to achieve hypothermia [10]. Professor Easmon (Figure 1) was the first dean of the University of Ghana Medical School; his grandfather J.F Easmon, a Ghanaian physician, coined the term blackwater fever to describe what was until then an enigmatic disease manifesting as a severe febrile illness accompanied by the passage of dark urine in patients suffering from malaria. Professor Easmon's team encountered stiff opposition to further development of open heart surgery in Ghana, the endeavour being considered a low priority for a country faced with a major burden of infectious diseases and malnutrition. Ghana's open heart program therefore faltered from such a bright early start due to lack of government commitment and poor staffing. Incessant military coups resulted in political instability that also contributed to demise of the cardiac program.

Professor Kwabena Frimpong-Boateng, another Ghanaian surgeon (Figure 1), returned to Ghana in 1989 after training at the Medizinische Hochschule Hannover, Germany. Kwabena Frimpong-Boateng, who received his basic medical training at the University of Ghana Medical School, had gained world-renown in Germany as one of the pioneers of the heart transplantation program in Hannover. Against staggering odds, he set up Ghana's National Cardiothoracic Center (NCTC) at the Korle Bu Teaching Hospital in 1989.

The efforts so far in these West African countries had been led by indigenous locally resident cardiothoracic surgeons who received their training from Europe and or America. In the last couple of years, an Italian group has led an effort at the Shisong Hospital in Cameroon to perform open-heart procedures. Reports from this mission [3], however, appears overzealous as claims have been made to the effect that this is the only center in west and central Africa performing open heart procedures. Recently, a coronary bypass procedure performed in this institution was also reported as the first of its kind in West/Central Africa [4]. The error in these reports [3, 4] is obvious from the current review.

## Ghana's National Cardiothoracic Center

**Figure 2:**
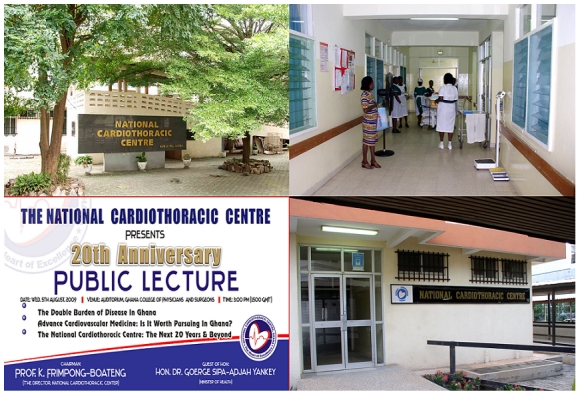
The National Cardiothoracic Center (2009): 20th anniversary

Cardiothoracic surgery in Ghana was revived when K. Frimpong-Boateng started work in 1989 at the Korle Bu Teaching Hospital in Accra. His effort to set up an autonomous center dedicated to cardiothoracic surgery was met with stiff opposition reminiscent of the Easmon era. With remarkable perseverance and leadership, he succeeded in setting up Ghana's NCTC which was officially commissioned on April 10^th^ 1992. In August of 2009, the NCTC celebrated 20 years of service (Figure 2) and used the occasion to outline to the Ghanaian public a strategic plan of expansion for the next 20 years.

The NCTC was set up with 3 major goals – service (in terms of addressing the cardiothoracic problems of the nation), training of cardiothoracic specialists, and research into the relevant cardiothoracic problems of the West African sub-region.

### Service and outcomes

**Figure 3:**
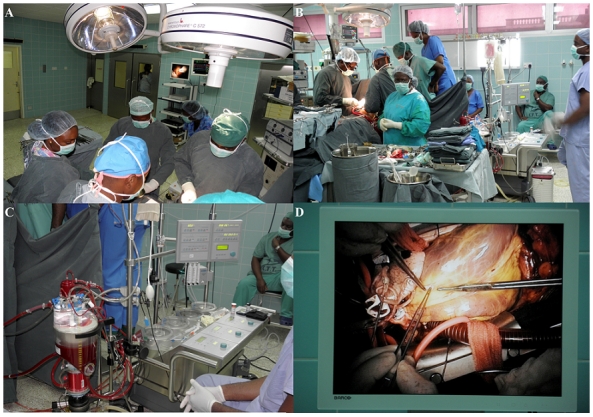
Coronary artery bypass in progress with remote liquid crystal display (LCD) for education

The courageous step taken by the government of Ghana and the initiative of Professor Frimpong-Boateng to establish the NCTC has resulted in a health institution that has had a tremendous impact on the health system of the country. The NCTC is a dedicated center with its own wards (30 beds), 2 operating theatres, a 6-bed intensive care unit, 6-bed high dependency unit, a dedicated laboratory, cardiac catheterization, radiology, echocardiography services, and a renal dialysis unit. The operating theatres have been equipped with in-light high definition (HD) operating room cameras for capturing video and still pictures for transmission and display on screen for education (Figure 3). A core team of 7 cardiothoracic surgeons is assisted by cardiologists, anesthetists, nephrologists, cardiovascular perfusionists, dedicated cardiovascular nurses, technologists, and other staff to run the center.

As at the beginning of 1995, the NCTC was attending to between 20-30 out-patients every week and had been performing between 100-120 surgical operations annually. By the year 2008, out-patient attendance had risen to 210 cases per week and surgical volume had gone up to 464 cases annually; roughly 25% of surgical cases annually are open-heart procedures. In a typical year, the commonest open heart procedures are those for rheumatic heart disease and congenital heart disease [11]. Coronary artery bypass procedures (Figure 3) are performed relatively infrequently compared to surgery for congenital heart disease and rheumatic heart disease.

Surgical outcomes at the NCTC are comparable to international standards and have improved over the years. Between 1991 and 2000, hospital mortality for mechanical valve replacement was 9.9% with actuarial survival of 85% at 5 years [12]. We recently reviewed our results for left heart mechanical valve replacement in children ≤18 years old [13]. We demonstrated an improvement in outcomes over the results of 2001 – hospital mortality had dropped to 5.3% and actuarial survival had improved to 94% at 15 years. Congenital heart disease is the next major diagnostic group seen at the NCTC. Adolescents and adults (>18 years old) with congenitally malformed hearts made up 23% of all congenital heart surgeries performed at our institution between 1993 and 2008 [2]. Hospital mortality was 3% for this patient population which consisted largely of patients with isolated septal defects of the atrial and ventricular septa, and Fallot's tetralogy. Results for congenital heart disease in children show a similar spectrum and outcome. Permanent complete heart block following congenital heart surgery is a morbidity concern in patients undergoing repairs involving procedures on the ventricular septum. Our institutional review of all patients who had intra-cardiac repair of congenital heart disease known to predispose to post-operative complete heart block from January 1993 to December 2008 has been reported elsewhere [14]. We demonstrated a risk of complete heart block of 1.3% for isolated ventricular septal defects, 5.5% for conotruncal defects of the Fallot type, and an overall risk of 2.5% (6 of 242 patients) [14].

Ghana's NCTC has served as a surgical hub for patients not only from Ghana but from the whole West African sub-region [2, 13]. Patients are referred regularly from Nigeria, the Gambia, Sierra Leone, Liberia, Togo, and others. The expatriate community and a large section of the diplomatic community have found in the NCTC an institution they can rely on in cases of emergency [15].

The economic impact of Ghana's NCTC has been enormous. In the past, the country could only sponsor less than a dozen citizens for cardiac surgery abroad on a yearly basis. About $50 000 per patient was spent, more in some cases. The establishment of the NCTC saw a drastic reduction in referrals abroad for cardiac surgery. In the period between 1996 and 1999, 650 patients underwent open heart surgery at the NCTC at an average cost of $6 000 per patient compared to the $50 000 some patients were paying for the same service in America, excluding air fares, accommodation, and related expenses. The estimated savings for the country during the period was in the region of $30 000 000 [15]. Additionally, the availability of a center with the capacity for high level invasive monitoring, emergency cardiac intervention and open cardiac surgery capability has had a major impact on the decision of potential investors who consistently assess the health care delivery system of a country before relocating high level manpower to a country considered to have positive prospects for investment capital.

### Training cardiovascular and thoracic specialists

The NCTC has been accredited by the West African College of Surgeons as a center of excellence for training cardiothoracic surgeons in the sub-region [16]. Five Ghanaian cardiothoracic surgeons have so far been trained, the first qualifying in 1999. All the locally trained Ghanaian surgeons are on staff at the NCTC presently. Another Ghanaian cardiothoracic surgeon trained in Cuba joined the NCTC in 2008. Several (>20) cardiothoracic surgeons have been trained from Nigeria, Togo, and Ethiopia. Ghana's NCTC has become a preferred destination for cardiothoracic residents seeking practical experience in cardiothoracic surgery before their final cardiothoracic fellowship examinations. The center has also boosted the undergraduate program of the University of Ghana Medical School by providing practical training to undergraduates for cardiology, cardiothoracic surgery, and cardiothoracic anesthesia.

**Figure 4:**
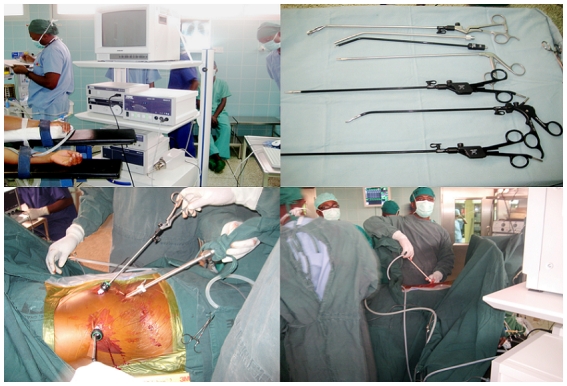
The video-assisted thoracoscopic surgery (VATS) service of the Ghana's National Cardiothoracic Center

With the current global emphasis on sub-specialization, the NCTC has aligned its training policies accordingly. The first locally-trained surgeon received further training in coronary artery surgery and adult cardiac surgery in Aalst (Belgium). With the help of donors, the NCTC acquired equipment for video-assisted thoracoscopic surgery (VATS) and later sponsored the training of two locally-trained Ghanaian cardiothoracic surgeons in Germany to run the VATS service. Our VATS service (Figure 4) has been in operation since 2008.

Recently, through fruitful collaborations with the Walter Sisulu Pediatric Cardiac Foundation based at the Netcare Sunninghill Hospital in Johannesburg, another locally-trained Ghanaian surgeon is near completion of a 2-year training in pediatric and congenital cardiac surgery. The center is putting together a team of trained specialists dedicated to pediatric and congenital heart surgery to run a dedicated pediatric cardiac surgery service. Our training philosophy is based on the conviction that improved results in cardiothoracic surgery require specialized teams dedicated to the care of specific patient populations.

#### Retention of trained staff

Exodus of trained staff from developing countries to Europe and America is a pervasive problem. This phenomenon referred to as the “brain drain” has been discussed in various forums. Studies in Ghana indicate a number of “push” and “pull” factors [17]. “Push” factors are those factors that occur within the country of origin, motivating professionals to leave. “Pull” factors on the other hand are the deliberate and/or unintended actions that attract health professionals originating from the recipient country's policies and actions. Examples of ‘Push’ factors include low remuneration, poor working conditions and low job satisfaction, political and ethnic problems as well as civil strife and poor security. The lack of technology and equipment to perform professional tasks for which staffs are trained will reduce job satisfaction. “Pull” factors on the other hand may arise because of increased demand for health professionals in developed countries with better remuneration. A complex combination of these “push-pull” factors results in the creation of certain gradients along which migration of health professionals occur. Important among these are income, opportunities for career advancement, social security and retirement benefits, job satisfaction, and governance gradients [17]. At the NCTC, “brain drain” of trained staff has not been experienced to a major degree. Some trained staffs have immigrated to developed countries but the majority, including all the trained cardiothoracic surgeons, have stayed and worked in Ghana. This is probably due to the fact that the leadership at the NCTC took advantage of the modifiable aspects of the “push-pull” factors to motivate trained staff to stay. The commitment to staff training and international exposure provided opportunities for career development and advancement. The provision of appropriate modern technology ensures that trained staffs have the equipment to perform what they have been trained to accomplish. Technical proficiency, international collaboration, involvement in relevant research and innovation allows for international recognition with one's peers that promote job satisfaction. Service to the international and expatriate community allowed the leadership of the center to invest in the provision of housing for its senior personnel so that staff did not have to worry about post-retirement accommodation. These efforts were given an additional boost in the late 1990s when the Government of Ghana improved service conditions and remuneration for medical staff across the country.

### Research in cardiothoracic surgery

The vast socio-economic and health needs of the African continent have contributed to relegating cardiovascular research in Africa into relative insignificance. Not surprisingly, a study on global cardiovascular research output showed that the cardiovascular research contribution of Africa in the number of published articles was the lowest of all continents at 0.3% [18]. The cardiovascular research output in Africa probably characterizes the practice of our specialty on the continent and brings to remembrance the words of Albert Starr– “…practicing surgery in an intellectual vacuum” [19]. Without the foresight afforded by relevant research, our progress is bound to be slow and our contribution to the global advancement of knowledge and innovation in cardiothoracic surgery will be slight. The NCTC has shown commitment to cardiothoracic research resulting in the publication of several papers in local and international peer-reviewed journals some of which have already been cited in this communication [2, 11, 13, 14]. Additionally, our team has reported several surgical innovations since the inception of the NCTC:

#### Sternocleidomastoid myocutaneous esophagoplasty

In 1994, Frimpong-Boateng described four cases of short segment non-malignant lesions involving the cervical esophagus following failed esophageal dilatation [20]. The patients were managed surgically using an innovative sternocleidomastoid myocutaneous flap as a patch to widen the stenotic esophageal segment. There were no operative deaths and no leakage of the repair. There was no stricture recurrence or ulceration of the patch after 0.5-6.0 years of follow-up. We are of the conviction that the simplicity and efficacy of this method as against other more extensive surgical procedures, should be enough reason to consider it as a viable alternative in intractable short segment stenosis of the cervical esophagus.

#### Cardiopulmonary bypass in sickle cell anemia without exchange transfusion

Our group is one of the few in the world that has reported successful cardiopulmonary bypass (CPB) in sickle cell disease (SCD) without preoperative exchange transfusion [21, 22]. The majority of cases of CPB in SCD (mostly sickle cell trait) have employed preoperative exchange transfusion to reduce the hemoglobin S fraction to <30% based on the assumption that such ‘dilution’ of hemoglobin S reduces perioperative complications attributable to SCD [23]. We are convinced that there is a lack of definitive control data to validate this practice. In Ghana, SCD is present in 2% of newborns and the hemoglobin S gene is present in 20% of individuals [23]. Our technique of CPB in SCD has been described elsewhere [21]. We reported successful mechanical mitral valve replacement without preoperative exchange transfusion in two patients both aged 12 years (hemoglobin SS) and tricuspid valve repair in a third patient aged 17 years (hemoglobin SC) in 2001 [24]. Our experience suggests that by meticulously avoiding hypoxia, acidosis, dehydration, and hypotension, CPB can be safely performed in SCD patients without preoperative exchange transfusion. Using the same precautions, systemic hypothermia may be employed together with cold cardioplegic arrest to obtain a quiet surgical field necessary for safe intra-cardiac procedures.

#### Trachea stabilization with autologous costal cartilage in acquired tracheomalacia

Post-operative tracheomalacia is a life threatening condition whose management is challenging. Suggested surgical procedures to manage the condition include tracheostomy, staged thyroid reductions and the use of intra- or extra-luminal stents. Our team reported in 2001, the successful management of this condition using autologous costal cartilage to support the tracheal wall [25].

#### Cardiopulmonary bypass in Jehovah's witnesses

The need to avoid transfusion of heterologous blood in Jehovah′s Witnesses presenting for open heart surgery prompted us to adopt innovative methods based on pre-operative administration of nutritional supplements, hematenics, erythropoietin, antimalarials and the modification of the extra-corporeal bypass circuit to allow successful cardiopulmonary bypass in these patients [26].

#### Colopharyngoplasty for intractable caustic pharyngoesophageal strictures

Surgical management of caustic strictures of the upper digestive tract poses difficult challenges because reconstruction above the cricopharyngeal junction interferes with the mechanisms of swallowing and respiration. We recently reported our experience and surgical technique of colopharyngeal reconstruction of the challenging subset of patients with severe diffuse pharyngoesophageal caustic strictures accompanied by upper airway obstruction [27]. We showed good results with the establishment of digestive tract continuity using a suprahyoid anastomotic technique. Rehabilitative training for deglutition was a universal requirement in the postoperative period to establish near-normal swallowing. We cautioned that concomitant tracheostomy portends a substantial long term mortality risk.

#### Pediatric cardiac surgery

Our collaboration with the Walter Sisulu Pediatric Cardiac Center for Africa resulted in several publications in the field of pediatric and congenital heart surgery. We have demonstrated the feasibility and safety of performing the primary arterial switch procedure for transposition of the great arteries with intact ventricular septum up to the first 10 weeks of life [28, 29]. Many centers around the world limit this procedure to the first 3 weeks of life due to concerns that the involuted left ventricle is unable to support a systemic circulation beyond the first 3 weeks of life; a two stage arterial switch procedure has hitherto been the preferred approach to the management of infants presenting beyond 3 weeks of life.

Our unique approach of optimum exposure of the subaortic region using the six-point hexagonal traction technique is in press [30].

Others include descriptions of extremely rare congenital heart defects – retro-esophageal aortic arch [31], double outlet right atrium, and isolation of the right subclavian artery [32].

## The future of cardiothoracic surgery in West Africa

The development of cardiothoracic surgery appears to closely parallel economic development. Roughly 42% of all cardiothoracic surgeons practise in North America and 32% in Europe. Africa has only 1% of the world's cardiothoracic surgeons at its disposal. There is only one cardiothoracic surgeon for 4 million inhabitants in Africa compared to 28 per million in Western Europe and North America [33]. As at 2009, Ghana's estimated population stood at 23.8 million with a Gross National Product (GNP) per capita of $1,500 [34]. Nigeria, ostensibly the most economically-endowed nation in the sub-region had an estimated population of 149.2 million and a GNP per capita of $2,300. The 2009 GNP of North America (population 307.2 million) was $47,000 and $34,800 in Germany (population 82.3 million). The density of cardiothoracic surgeons very closely follows the distribution of GNP, an indication of the heavy financial outlay required to establish the infrastructure and practice of modern cardiothoracic surgery [33]. Economic development should therefore lead to a commensurate development of cardiothoracic surgery in the sub-region. The critical link between political stability and economic growth draws a secondary link between political stability and the regional/national development of cardiothoracic surgery.

The ability of patients to pay for the service is inexorably linked to both economic development and national development in cardiothoracic surgical services. All over the world, costs relating to cardiothoracic surgery rank high among healthcare costs. In the developed world, the most viable model of meeting this cost has been through medical insurance. For much of West Africa, there is no viable insurance scheme that covers cardiothoracic surgery; patients must pay for the service (if available) ‘out of pocket’, a situation that often takes years to materialize. Philanthropy and donor support are useful but constitute no lasting solution. The model in Ghana has been helpful – the Ghana Heart Foundation, a non-governmental organization set up by Professor Frimpong-Boateng defrays some of the cost for Ghanaian patients requiring open-heart surgery. The availability of health insurance cover for open-heart surgery will ostensibly be a better model but depends on the ability of workers to pay the required premiums. Without consistent economic growth, this shall remain a dream. The urgent need for economic growth and fiscal prudence in West Africa is thus a critical health issue. The impact of such “push” factors on the “brain drain” can only continue to worsen if our economies do not improve.

Our experience in Ghana confirms these observations. Ghana has enjoyed a reasonably stable political climate and a modest economic growth since 1990, just about the time the NCTC was set up. Further growth in our cardiac program can only be sustained if we manage our economy prudently and make sound healthcare policy decisions in terms of cardiovascular disease prevention, infrastructural layouts, staff training, and retention.

### 

#### Is investment in cardiothoracic surgery justifiable for poor nations?

The question is often asked whether it is justifiable for poor countries such as those in West Africa to invest in cardiothoracic surgical healthcare delivery. In most West African countries, the argument goes; infectious disease and malnutrition constitute more pressing health needs than cardiac and thoracic pathologies. In Ghana for instance, 42.1% of all deaths in 2009 were due to malaria, diarrheal diseases, HIV/AIDS, and respiratory infections. Congenital anomalies and rheumatic heart disease together accounted for only 1.8% of all deaths [34]. How then do we justify the heavy financial outlay required to set up and run a modern cardiothoracic facility when we have such a huge problem with infectious diseases? What is often overlooked is that infectious disease is primarily not a medical problem but the reflection of a social system that has failed to institute the appropriate environmental sanitation and personal hygiene practices. Infectious diseases need to be eradicated through improved environmental sanitation and personal hygiene. This applies to rheumatic heart disease as well. On the other hand, apart from birth control, relatively little can be done to reduce the incidence of congenital heart defects. The facts notwithstanding, every patient has the right to be diagnosed and treated regardless of diagnosis or statistics. With a stable incidence of congenital heart disease of 0.8% of live births around the world, 2009 UNICEF data [35] for annual births and infant mortality rate would predict a congenital heart disease burden in Ghana of 5,840 new cases per year. Using the same principle, the estimate for the West African sub-region is roughly 89 000 new cases per year. Nigeria, Africa's most populous nation accounts for nearly half the figure (44,464 new cases) for the entire sub-region. Certainly, we cannot neglect to care for such numbers on the basis of economics. It must not be overlooked that from our experience in Ghana, roughly 70% of congenital heart cases are simple defects [36] that are curable by surgery with very low reoperation rates. Furthermore, we have shown in Ghana that a good cardiothoracic facility elevates the standard of healthcare in a developing country, a positive consideration for winning investor capital. Importantly, health systems cannot be built ‘brick-upon-brick’ like a house, stacking one disease on top of the other. The comment of Nigeria's President Olusegun Obasanjo is instructive: “We cannot afford to say ‘we must tackle the other diseases first—HIV/AIDS, malaria, tuberculosis—then we will deal with chronic disease; if we wait even 10 years we will find that the problem is even larger and more expensive to address” [37]. For these reasons, we believe investment in cardiothoracic surgical healthcare is sound and just. Collaboration with established centers is encouraged in the development of the specialty in our sub-region. If the West African sub-region is to progress in the field of cardiothoracic surgery, economic development is a sine qua non. Trained cardiothoracic specialists with visionary leadership will maximize what is made available by a strong national economy.

## Conclusion

The cardiothoracic surgical landscape in West Africa is neither ‘blank’ nor ‘infantile’. Several West African countries have documented reputable historical accounts of open-heart surgery spanning decades. The Ghanaian experience of open-heart surgery demonstrates the critical link between a stable political climate, economic growth, good leadership with prudent fiscal management, and a sustained growth in cardiothoracic surgical services. The future of cardiothoracic surgery in West Africa will be determined by political stability and sustained economic growth in the sub-region. Visionary and competent cardiovascular specialists are needed to lead the way in training, infrastructural layouts, research, innovation, and technological advancement. A viable cardiac program is best developed by a senior surgeon who is locally-based and takes up the training and supervision of younger indigenous surgeons who are motivated to stay and contribute their quota to national development in this specialty.
